# Altered regional homogeneity in Parkinson’s disease with mild cognitive impairment: a resting-state fMRI study

**DOI:** 10.3389/fpsyg.2026.1816245

**Published:** 2026-05-08

**Authors:** Xudong Zhu, Jiaojiao Zhou, Qiancheng Wei, Hong Pu, Yan Chen, Xiaowei Zhu, Bin Li, Shihua Liu

**Affiliations:** 1 Department of Neurology, Suzhou Hospital of Anhui Medical University, Suzhou, Anhui, China; 2 Department of Radiology, Suzhou Hospital of Anhui Medical University, Suzhou, Anhui, China

**Keywords:** cognitive impairment, neuroimaging biomarkers, Parkinson’s disease, regional homogeneity, resting-state functional MRI

## Abstract

**Background:**

To explore changes in regional homogeneity (ReHo) of brain function in patients with Parkinson’s disease with mild cognitive impairment (PD-MCI) using resting-state functional magnetic resonance imaging (rs-fMRI), and to analyze the correlation between ReHo values in differential brain regions and cognitive function, so as to identify potential neuroimaging biomarkers for PD-MCI.

**Methods:**

A total of 43 patients with PD were enrolled and divided into the PD-MCI group (*n* = 23) and the PD with normal cognitive function (PD-NC) group (*n* = 20). Additionally, 20 healthy controls (HC) were recruited. The ReHo method was used to evaluate differences in functional consistency of brain regions, and the Montreal Cognitive Assessment (MoCA) scale was employed to assess cognitive function. Further, a correlation analysis was performed between the ReHo values of each brain region and MoCA scores in patients with PD-MCI and PD-NC and the receiver operating characteristic (ROC) curve was used to evaluate the discriminative efficacy of these brain regions for PD-MCI.

**Results:**

Compared with the PD-NC group, the PD-MCI group showed significantly decreased ReHo values in the right middle frontal gyrus and right superior frontal gyrus (*t* = −3.3113, −4.1326, both *p* < 0.05), and significantly increased ReHo values in the right cerebellar lobule VIII, left inferior temporal gyrus, and right fusiform gyrus (*t* = 4.9059, 2.9759, both *p* < 0.05). MoCA scores were negatively correlated with ReHo values in the right cerebellar lobule VIII, left inferior temporal gyrus, and right fusiform gyrus (*r* = −0.5597, −0.6239, *p* < 0.05), and positively correlated with ReHo values in the right middle frontal gyrus and right superior frontal gyrus (*r* = 0.5690, 0.5296, *p* < 0.05). ROC analysis showed that the ReHo values of the right cerebellar area VIII and the left inferior temporal gyrus had high efficacy in distinguishing PD-MCI from PD-NC, with an area under the curve (AUC) of 0.9326.

**Conclusion:**

Functional abnormalities in brain regions including the right middle frontal gyrus, right superior frontal gyrus, right cerebellar lobule VIII, left inferior temporal gyrus, and right fusiform gyrus are closely associated with the occurrence and development of PD-MCI. ReHo indicators in these regions are promising potential neuroimaging biomarkers for auxiliary diagnosis and monitoring the progression of PD-MCI.

## 1 Introduction

Parkinson’s disease (PD) is the second most common neurodegenerative disease after Alzheimer’s disease (Watanabe et al., 2024). Cognitive impairment, as one of the most prevalent non-motor symptoms, has garnered increasing attention. Longitudinal and cross-sectional studies have reported that approximately 20–53% of PD patients have concomitant mild cognitive impairment (MCI). The variability in these estimates may be attributed to methodological differences, such as diagnostic criteria for PD-MCI, sample size, disease duration, disease severity, medication status, and educational level. PD-MCI is a transitional stage between PD with normal cognitive function (PD-NC) and PD with dementia (PDD). Most PD-MCI patients progress to PDD within 3–5 years, with an annual conversion rate of approximately 10–15% (Lee et al., 2014; Pfeiffer et al., 2014; Hobson and Meara, 2015), which seriously affects patient prognosis. However, the pathogenesis of PD-MCI remains unclear.

In recent years, resting-state functional magnetic resonance imaging (rs-fMRI), a non-invasive imaging technique, has been widely used in the diagnosis and research of PD due to its ability to reflect constant brain activity based on blood oxygen level-dependent low-frequency fluctuations (Liu et al., 2025; Song et al., 2025). Regional homogeneity (ReHo) is a commonly used fMRI method, whose theoretical basis lies in the high temporal sequence consistency among individual voxels within the same brain region during activation. The Kendall’s coefficient of concordance (KCC) calculated using the similarity of temporal sequences among voxels reflects the temporal synergy of neuronal activity (Hu et al., 2019). Increased or decreased ReHo values indicate enhanced or weakened local functional integration of neuronal activity in the corresponding brain region (Liu et al., 2019), respectively. Compared with functional connectivity analysis that focuses on long-range connections, ReHo does not require predefining seed points, thus avoiding subjective selection bias, and is robust to both temporal and spatial noise (Shen and Xu, 2022). These methodological advantages make ReHo a valuable tool for understanding abnormal local information processing efficiency related to diseases.

In the field of PD research, rs-fMRI has initially revealed extensive resting-state network disruptions underlying motor symptoms, such as altered functional connectivity in the default mode network (DMN)(Tessitore et al., 2019), salience network, dorsal attention network, and sensorimotor network. These network abnormalities are considered closely related to non-motor symptoms of PD, including cognitive impairment and emotional disorders (Shen and Xu, 2022). However, studies specifically investigating changes in the synchrony of local spontaneous neural activity in PD-MCI patients are limited, and existing research results are inconsistent. Some studies have reported decreased ReHo in higher cognitive-related brain regions such as the prefrontal cortex and temporoparietal junction in PD-MCI patients (Guo et al., 2021), suggesting local functional disintegration; other studies have observed increased ReHo in regions such as the precuneus and superior parietal lobule (Li et al., 2020), which may reflect compensatory mechanisms. These inconsistencies may stem from issues such as participant heterogeneity and differences in research methods. Whether ReHo alterations in PD-MCI exhibit disease-specific patterns remains debated, with existing evidence largely derived from small samples or studies that inadequately control for motor symptom confounds; the distinctive ReHo signatures in PD-MCI have not been systematically characterized; and the correlation between ReHo abnormalities and cognitive severity in PD-MCI remains poorly elucidated.

This study aims to employ rs-fMRI using ReHo analysis, in conjunction with strictly matched cohorts of PD-MCI and PD-NC patients, to investigate: (1) Whether there exist characteristic brain regions exhibiting altered ReHo in PD-MCI; (2) Whether ReHo values in these differential brain regions correlate with the severity of cognitive impairment in PD patients; (3) The diagnostic efficacy of ReHo value changes in specific brain regions for PD-MCI. The study aims to provide imaging evidence for the pathophysiological mechanisms underlying PD-MCI and to develop an objective ReHo-based diagnostic biomarker.

## 2 Materials and methods

### 2.1 Participants

This study collected data from 43 patients with PD treated at Suzhou Hospital Affiliated to Anhui Medical University from January 2022 to June 2024, as well as 20 healthy controls (HC) matched for age, sex, and years of education.

Inclusion criteria for PD patients: Meeting the 2015 PD diagnostic criteria of the International Parkinson and Movement Disorder Society (MDS) (Postuma et al., 2015); all participants were right-handed; aged 40–80 years. Exclusion criteria: Atypical parkinsonism (e.g., progressive supranuclear palsy, corticobasal degeneration, dementia with Lewy bodies); secondary parkinsonism (e.g., vascular parkinsonism, drug-induced parkinsonism); history of traumatic brain injury, encephalitis, definite cerebral infarction, cerebral hemorrhage, or documented mental illness; severe underlying cardiovascular, cerebral, renal diseases, or malignant tumors.

The diagnosis of PD-MCI is based on the first-level MDS diagnostic criteria for PD-MCI (Litvan et al., 2012), with reference to the validated criteria for the low-educated population in China. A cut-off score of 20 points is used for the diagnosis of PD-MCI, which is applicable to PD patients with 6 years of education or less (Chen et al., 2016). The above inclusion criteria are integrated: (1) History of PD; (2) MoCA score < 20; (3) Cognitive impairment reported by the patient or informant, without significant impact on work and daily life. PD patients were divided into the PD-MCI group (*n* = 23) and the PD-NC group (*n* = 20). All diagnoses were confirmed by at least two neurologists specializing in movement disorders.

All patients and healthy controls were recruited from Suzhou Hospital of Anhui Medical University and signed written informed consent forms. The study was approved by the Ethics Committee of Suzhou Hospital of Anhui Medical University (Approval No.: A2023026).

### 2.2 Clinical characteristic measurement

General information of patients, including age, gender, and educational level, was collected. All participants in the PD group underwent MoCA scale assessment for cognitive function, Hoehn-Yahr (H-Y) staging for disease severity, and the Movement Disorder Society-sponsored revision of the Unified Parkinson’s Disease Rating Scale Part III (UPDRS III) for motor function evaluation.

### 2.3 Image data acquisition

Prior to neuroimaging, all participants underwent a > 12-h withdrawal from oral antiparkinsonian medications. Scanning was performed on a Philips Ingenia 3 T MRI system equipped with a standard head coil. Subjects were positioned supine with head immobilization using foam padding. During acquisition, participants were instructed to maintain rest with eyes closed, avoiding intentional cognitive or motor activities. Structural imaging: High-resolution T1-weighted volumes were acquired via 3D T1W-TFE sequence (Parameters: repetition time (TR) = 6.6 ms, echo time (TE) = 3 ms, flip angle (FA) = 12°, number of slices = 170, slice thickness = 1 mm, slice gap = 1 mm, field of view (FOV) = 240 × 240 mm^2^, matrix size = 512 × 512, and voxel size = 0.5 × 0.5 × 1 mm^3^). Functional imaging: Resting-state fMRI data were obtained using gradient-echo EPI (8-min duration; Parameters: TR = 2000 ms, TE = 30 ms, FA = 90°, number of slices = 33, slice thickness = 3.5 mm, slice gap = 0.7 mm, FOV = 224 × 224 mm^2^, matrix size = 128 × 128, and voxel size = 1.75 × 1.75 × 4.2 mm^3^).

### 2.4 Data processing and ReHo index calculation

rs-fMRI data were preprocessed using REST plus v1.27^1^. The preprocessing pipeline comprised: (1) Initial volume removal: Exclusion of the first 10 time points to achieve longitudinal magnetization equilibrium and mitigate scanner acclimatization effects. (2) Slice-timing correction: Temporal realignment for inter-slice acquisition delay compensation. (3) Motion correction: Rigid-body realignment to the first volume, with subjects excluded if exhibiting >3 mm maximum translation or >3° rotation. (4) Spatial normalization: Coregistration to T1-weighted structural images, followed by tissue segmentation and nonlinear warping to the Montreal Neurological Institute (MNI) template via deformation fields. (5) Linear detrending: Elimination of signal trends associated with scanner drift artifacts. (6) Nuisance regression: Incorporation of covariates including Friston-24 motion parameters, cerebrospinal fluid (CSF), and white matter signals. (7) Bandpass filtering: Frequency-based noise reduction (0.01–0.08 Hz) to suppress low-frequency drifts and physiological high-frequency noise.

ReHo analysis was performed using RESTplus v1.27. ReHo values were obtained by calculating the KCC of the time series of a given voxel and its 26 nearest neighboring voxels. For standardization, the ReHo value of each voxel was divided by the global average ReHo value. The standardized ReHo images were smoothed using a Gaussian kernel with a full width at half maximum (FWHM) = 4 mm.

### 2.5 Statistical analysis

Demographic and clinical data were analyzed using SPSS (v27.0.1) and GraphPad Prism (v10.0). The Shapiro–Wilk test was used to assess the normality of continuous variables. (1) Normally distributed data were presented as the mean ± standard deviation. One-way analysis of variance (ANOVA) was used for comparisons among three groups, and the independent-samples *t-*test was used for comparisons between two groups. (2) Non-normally distributed data were presented as the median (interquartile range). The Kruskal-Wallis *H* test was used for multiple-group comparisons, and the Mann–Whitney *U* test was used for comparisons between two independent groups.(3) Categorical variables were presented as frequencies (percentages) and analyzed using the chi-square test. A two-sided *p*-value < 0.05 was considered statistically significant.

Neuroimaging analysis: Statistical analysis of ReHo maps was performed using the REST toolbox. (1) A voxel-wise one-way analysis of covariance (ANCOVA) was performed to detect significant intergroup differences in ReHo among the PD-MCI, PD-NC, and HC groups, with sex, age, years of education, UPDRS III score, H&Y stage, and head motion parameters as covariates. Multiple comparisons were corrected using the Gaussian Random Field (GRF) theory. (2) *Post-hoc* two-sample *t*-tests were performed on significant clusters to clarify intergroup differences (PD-MCI vs. HC, PD-NC vs. HC, PD-MCI vs. PD-NC). (3) Significant brain regions were labeled according to the Automated Anatomical Labeling 3 (AAL3) brain atlas (Rolls et al., 2020), and their MNI coordinates, cluster size (voxels), and peak *t*-values were recorded.

Correlation analysis and statistical validation: A mask file was created based on the statistically significant brain regions identified from the ANOVA-corrected results. Subsequently, the mean ReHo value within each mask was extracted, yielding one average ReHo value per subject for each significant cluster. For the PD-MCI and PD-NC groups, Spearman correlation analysis was performed between the mean regional homogeneity values of each significant brain region cluster and the MoCA scores of each group. GRF theory correction addressed multiple comparisons (voxel *p* < 0.01, cluster *p* < 0.05, two-tailed). To evaluate the discriminative power between PD-MCI and PD-NC groups, receiver operating characteristic (ROC) curve analysis was employed, with diagnostic accuracy quantified by the area under the curve (AUC). Statistical significance was defined as *p* < 0.05 (two-tailed).

## 3 Results

### 3.1 Demographic and clinical characteristics

There were no statistically significant differences in gender, age, or educational level among the PD-MCI group, PD-NC group, and HC group. No significant differences in H&Y staging or UPDRS III scores were observed between the PD-MCI and PD-NC groups. The MoCA score in the PD-MCI group was significantly lower than that in the PD-NC group, with a statistically significant difference (*t* = −5.612, *p* < 0.001) (Table 1).

**Table 1:** Evaluation results of demographic and clinical data in three groups.

Demographic and clinical data	PD-MCI group	PD-NC group	HC	*t/H/X^2^ /Z*	*p-*value
	(*n* = 23)	(*n* = 20)	(*n* = 20)		
Age/years	67 (58.00,71.00)	65 (48.50,71.00)	61 (56.25,68)	1.474^a^	0.479
Gender Male/Female	14/9	7/13	12/8	3.552^b^	0.166
Years of education	6 (0.00,9.00)	6 (6.00,9.00)	9 (0.00,11.25)	3.378^a^	0.185
UPDRS-III score	33.1 ± 15.3	30.5 ± 6.1	NA	0.718^c^	0.477
H&Y stages	2 (1.50,3.00)	2 (1.12,2.50)	NA	-1.067^d^	0.286
MoCA score	15 (8.00,18.00)	24.5 (22.00,27.75)	NA	-5.612^d^	<0.001

^a^ Kruskal-Wallis *H* test; ^b^ *x*^2^ test; ^c^ two independent samples *t-*test; ^d^ Mann–Whitney *U* test.

### 3.2 Intergroup differences in ReHo

#### 3.2.1 Overall differences among the three groups (ANCOVA results)

**Figure 1:**
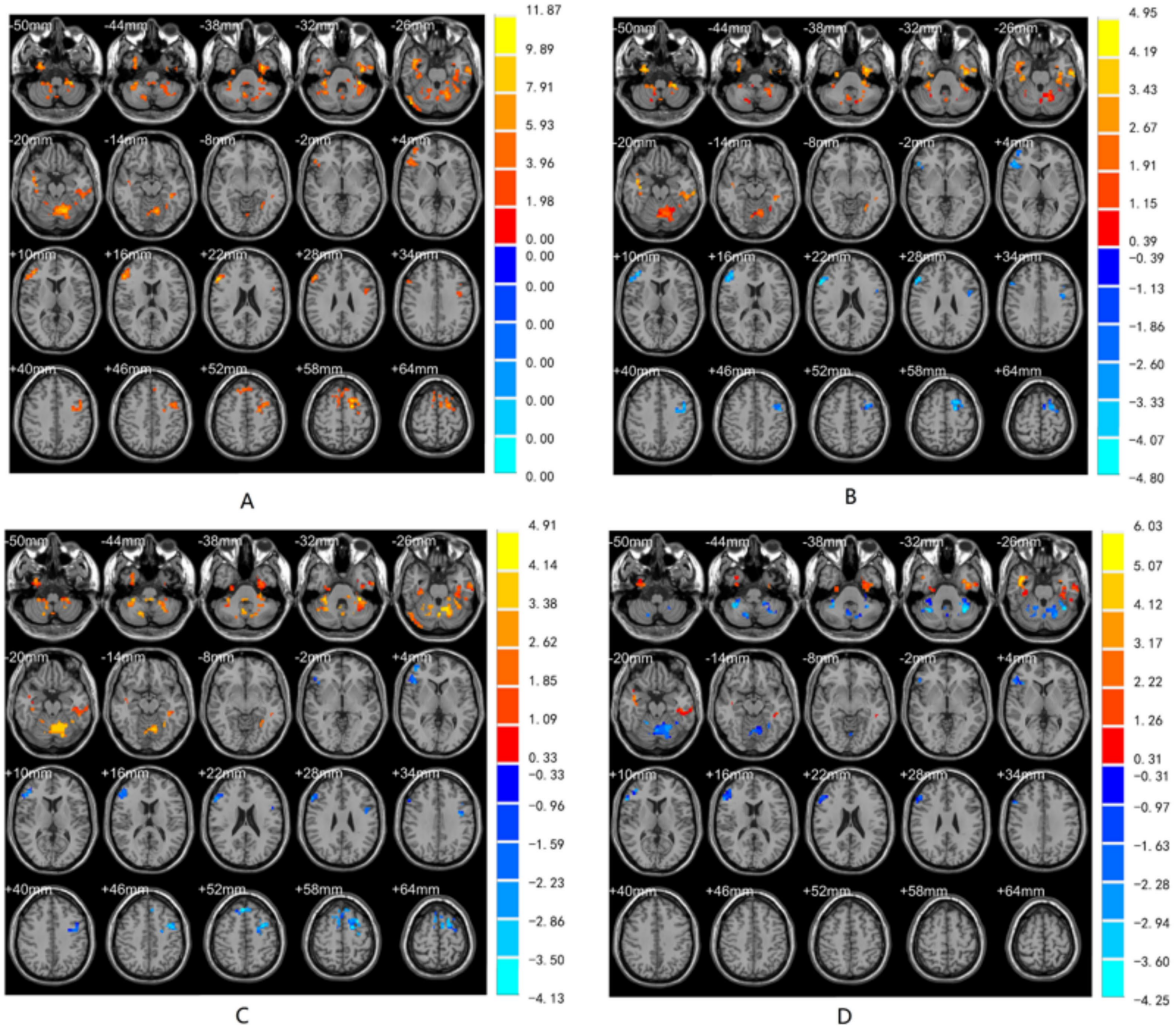
Slice view: **(A)** Brain regions with significant differences in ReHo values. **(B)** Brain regions showing differences between PD-MCI and HC. **(C)** Brain regions showing differences between PD-MCI and PD-NC. **(D)** Brain regions showing differences between PD-NC and HC. The numerical values on the right side of **(A)** represent the *F*-values from the ANCOVA among the three groups, while the values in **(B,C,D)** represent the *t*-values from *post-hoc* tests. Warm-colored areas indicate brain regions with significantly increased ReHo values, whereas cool-colored areas indicate the opposite (corrected for multiple comparisons using GRF, voxel-level *p* < 0.01, cluster-level *p* < 0.05).

ANCOVA showed significant differences in ReHo values among the three groups, mainly concentrated in the left inferior temporal gyrus, right fusiform gyrus, right inferior temporal gyrus, right middle temporal gyrus, right inferior frontal gyrus, and left superior frontal gyrus (Figure 1A).

#### 3.2.2 Specific alterations: PD-MCI vs. HC

Compared with the HC group, the PD-MCI group showed significantly increased ReHo values in the right cerebellar lobule VI, left inferior temporal gyrus, right fusiform gyrus, right middle temporal gyrus, and left superior temporal gyrus, and significantly decreased ReHo values in the left inferior frontal gyrus triangular part and left superior frontal gyrus (Table 2 and Figure 1B).

**Table 2:** Brain regions with significant differences in ReHo between group.

Cluster_ID	Brain regions (ALL 3)	Cluster size	Peak MNI coordinates			*t* value	*P* value
			X	Y	Z		
PD-NC vs HC							
1	Cerebellum_VI_L	851	−39	−51	−27	−4.255	0.0013
2	Fusiform_R	212	42	3	−24	6.025	<0.0001
2	Temporal_Inf_R						
2	Temporal_Mid_R						
3	Cerebellum_CrusI_R	173	36	−54	−33	−3.669	0.0007
4	Frontal_Inf_Tri_R	210	57	27	12	−3.4699	0.0013
PD-MCI vs HC							
1	Cerebellum_VI_R	637	36	−36	−36	3.3047	0.0019
2	Temporal_Inf_L	496	−30	−6	−42	4.9514	<0.0001
3	Fusiform_R	247	30	−6	−51	4.0807	0.0002
3	Temporal_Inf_R						
3	Temporal_Mid_R						
4	Frontal_Inf_Tri_R	249	54	27	21	−4.8039	<0.0001
5	Frontal_Sup_L	322	−18	0	57	−4.2162	0.0001
PD-MCI vs PD-NC							
1	Temporal_Inf_L	1,396	−15	−54	−27	4.9059	<0.0001
1	Cerebellum_VIII_R						
2	Fusiform_R	152	30	−3	−45	2.9759	0.0045
3	Frontal_Mid_R	226	39	54	6	−3.3113	0.0020
4	Frontal_Sup_Medial_R	509	6	33	51	−4.1326	0.0017

AAL 3, Automated Anatomical Labeling 3; MNI, Montreal Neurological Institute.

#### 3.2.3 Direct comparison: PD-MCI vs. PD-NC

Compared with the PD-NC group, the PD-MCI group showed significantly decreased ReHo values in the right middle frontal gyrus and right superior frontal gyrus, and significantly increased ReHo values in the left inferior temporal gyrus, right cerebellar lobule VIII, and right fusiform gyrus (Table 2 and Figure 1C).

#### 3.2.4 Specific alterations: PD-NC vs. HC

Compared with the HC group, the PD-NC group showed significantly increased ReHo values in the right inferior temporal gyrus, right fusiform gyrus, and right middle temporal gyrus, and significantly decreased ReHo values in the left cerebellar lobule VI, right cerebellar peduncle I, and right inferior frontal gyrus triangular part (Table 2 and Figure 1D).

### 3.3 Correlation analysis between altered ReHo values and MoCA scores

**Figure 2:**
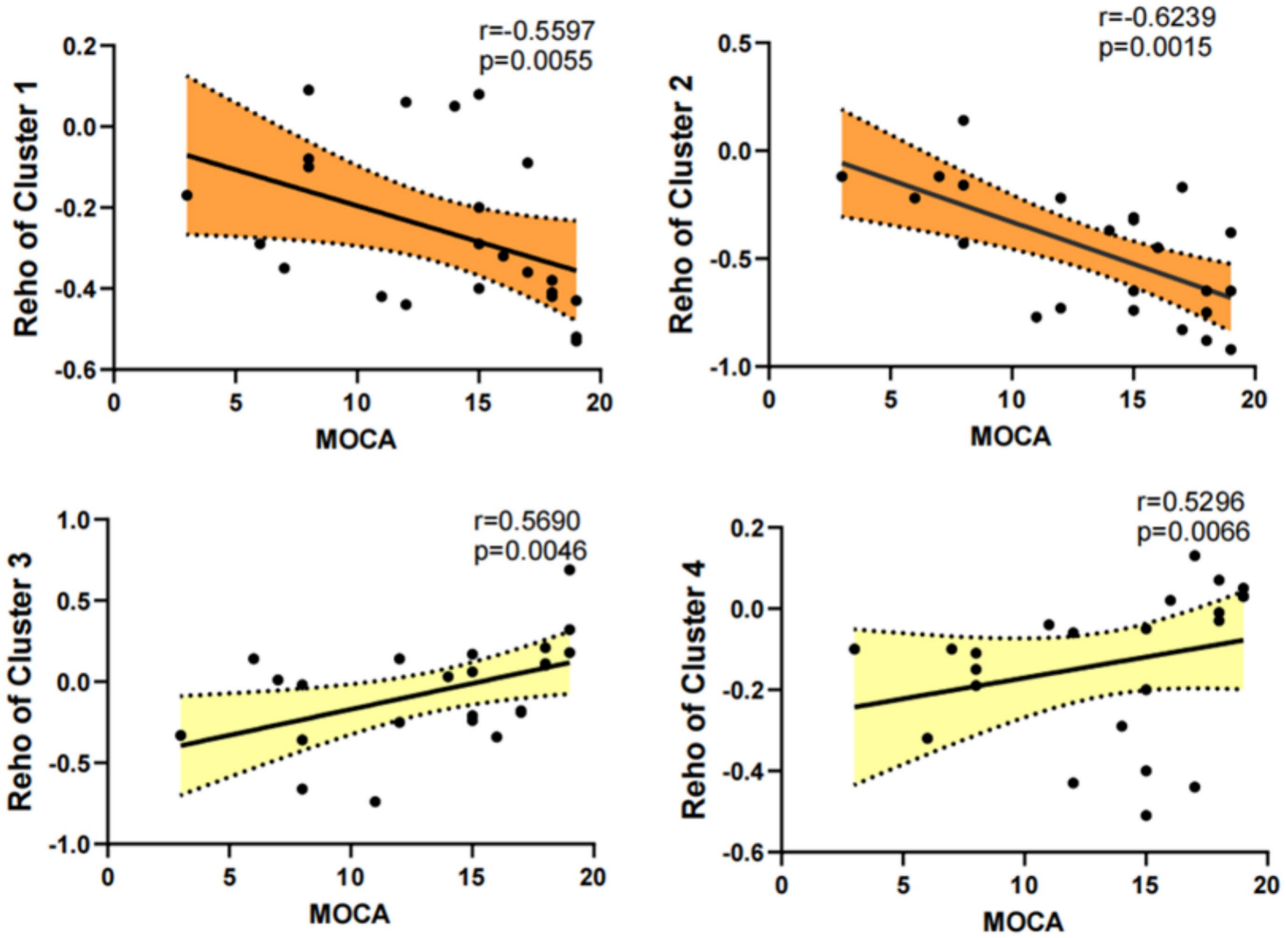
The ReHo values of Cluster 1 (*r* = −0.5597) and Cluster 2 (*r* = −0.6270) were significantly negatively correlated with MoCA scores in the PD-MCI group (*p* < 0.05), while the ReHo values of Cluster 3 (*r* = 0.5690) and Cluster 4 (*r* = 0.5496) were significantly positively correlated with MoCA scores (*p* < 0.05).

In the PD-MCI group, MoCA scores were significantly negatively correlated with ReHo values in the left inferior temporal gyrus, right cerebellar lobule VIII (*r* = −0.5597), and right fusiform gyrus (*r* = −0.6270) (*p* < 0.05), and significantly positively correlated with ReHo values in the right middle frontal gyrus (*r* = 0.5690) and right superior frontal gyrus (*r* = 0.5496) (*p* < 0.05). In the PD-NC group, the correlations between MoCA scores and ReHo values in each brain region did not reach statistical significance (all *p* > 0.05) (Figure 2).

### 3.4 Diagnostic efficacy of ReHo values in distinguishing PD-MCI

**Figure 3:**
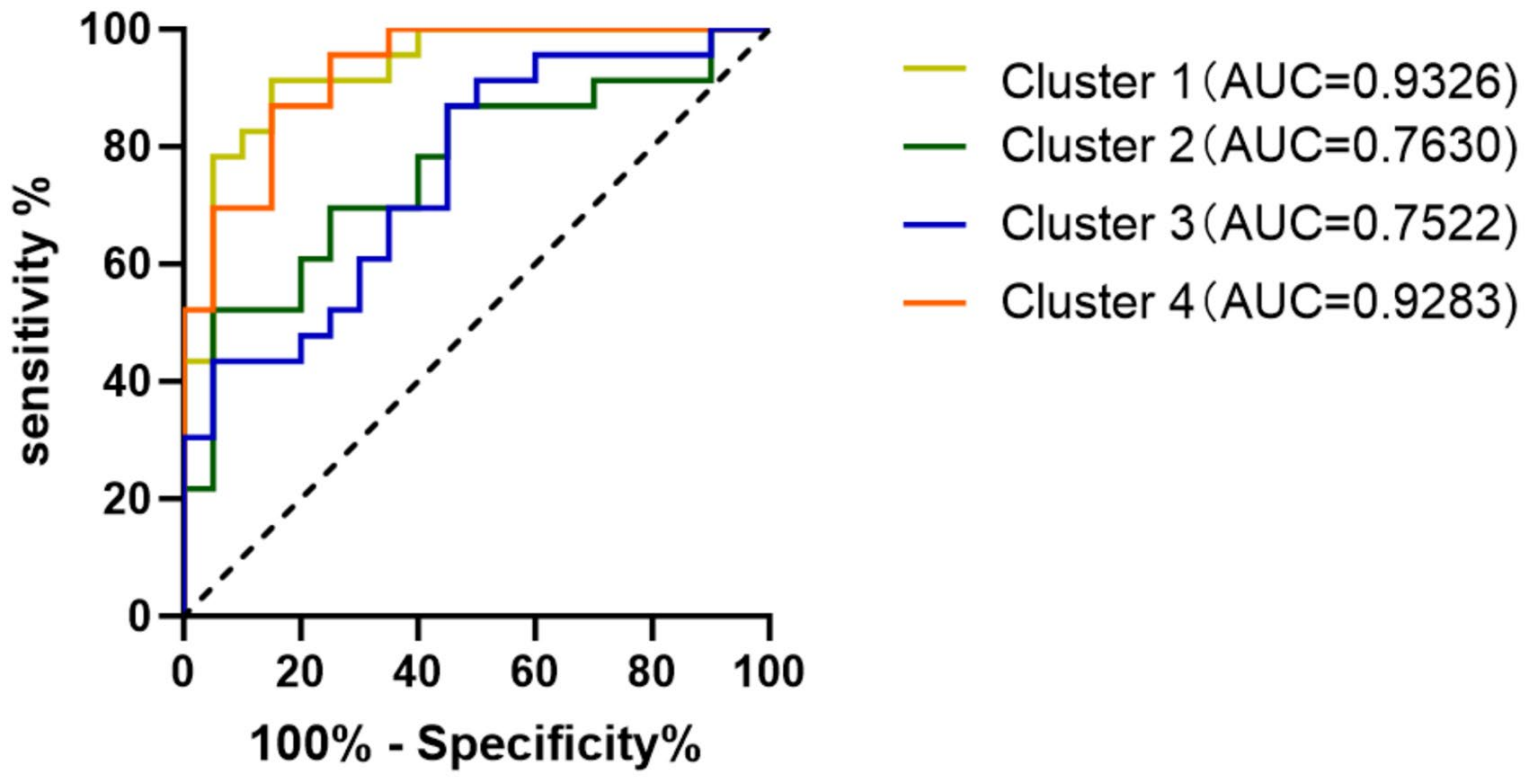
ROC curve analysis this analysis compares the diagnostic efficacy of ReHo from Cluster 1, Cluster 2, Cluster 3, and Cluster 4 in distinguishing PD-MCI from PD-NC. Cluster 1 (left inferior temporal gyrus, right cerebellar area VIII) shows the highest AUC of 0.9326.

Receiver Operating Characteristic (ROC) curve analysis showed that the ReHo values of the brain regions identified in the direct comparison between PD-MCI and PD-NC all have the ability to distinguish PD-MCI. Among them, the ReHo values of the right cerebellar area VII and the left inferior temporal gyrus showed the best diagnostic performance, with a high Area Under the Curve (AUC) of 0.9326 (Figure 3).

## 4 Discussion

This study adopted rs-fMRI combined with ReHo analysis to investigate the characteristics of brain functional activity in PD-MCI patients. The results demonstrated significant alterations in ReHo values in the right middle frontal gyrus, right superior frontal gyrus, right cerebellar lobule VIII, left inferior temporal gyrus and right fusiform gyrus in PD-MCI patients, and these changes were significantly correlated with the severity of PD-MCI symptoms, suggesting that these ReHo indicators may serve as potential neuroimaging biomarkers for PD-MCI. This spatial distribution pattern of “hypofunction in the anterior region and compensatory enhancement in the posterior region and cerebellum” provides new imaging evidence for understanding the neuropathological mechanisms of PD-MCI and reveals the complex characteristics of brain function reorganization during the occurrence of cognitive impairment.

The prefrontal cortex, composed of the middle frontal gyrus and superior frontal gyrus, plays a pivotal role in the neural regulatory network and acts as the core hub of high-level cognitive functions in the human brain. Recent studies have shown that the prefrontal cortex is extensively involved in motor control, decision-making and multimodal cognitive integration (Yu et al., 2022), and serves as an important node in core brain functional networks such as the DMN and executive control network (ECN) (Zhan et al., 2018; Martín-Bastida et al., 2021; Yuan et al., 2021). The DMN is a complex network consisting of several brain regions with temporal correlation that maintain normal metabolic activity when the human brain is in a resting state, and it is responsible for goal-free thinking processes such as self-introspection and emotional processing (Cremona et al., 2025). The ECN refers to a cortical network that supports sustained attention and working memory, and is involved in a variety of high-level cognitive tasks (Bi et al., 2022). The “dual syndrome hypothesis” proposed by Kehagia and colleagues further elaborates the pathological mechanism of cognitive impairment in PD (Ekman et al., 2014): on the one hand, damage to the dopamine-mediated frontostriatal circuit leads to executive dysfunction; on the other hand, impairment of the non-dopaminergic system dominated by the cholinergic pathway affects posterior cortical brain regions including the occipital lobe, cerebellum and temporal lobe, resulting in multidimensional cognitive decline in visual–spatial perception, memory encoding and attention maintenance. The significantly decreased ReHo values in the prefrontal lobe regions of PD-MCI patients observed in this study may be secondary to the loss of nigrostriatal dopaminergic projections, which alters brain metabolism and neural network patterns, ultimately leading to executive dysfunction. In addition, the prefrontal lobe serves as the core center for emotional regulation and cognitive control. Patients with PD-MCI often exhibit abnormal negative emotions, which can impair local neural synchrony through the limbic-prefrontal circuit (Liu et al., 2022). Therefore, the decreased ReHo in the prefrontal lobe of patients in this group is not only attributed to damage to the dopaminergic pathway but also related to the loss of emotional regulation control, both of which jointly accelerate the decline of cognitive function.

The left inferior temporal gyrus belongs to the high-order association cortex and is closely associated with emotional processing, semantic memory and social cognition (Sun et al., 2022). Abnormal functional activation of the temporal lobe cortex is commonly observed in patients with Alzheimer’s disease and other types of cognitive impairment (Saggu et al., 2025), which may reflect compensatory enhancement or pathological disorders in the brain’s processing of cognition-related information. In PD patients, the formation of Lewy bodies can also involve the temporal lobe, thereby impairing high-level cortical functions such as cognitive information integration and emotional regulation. Numerous studies have indicated that temporal lobe atrophy and reduced metabolism are common neuroimaging markers during the progression of PD to PDD (Decourt et al., 2021; Saha and Banerjee, 2021). The increased ReHo value in the left inferior temporal gyrus observed in this study may have two implications: on the one hand, to maintain the functions of visual object recognition and semantic processing, neurons in this region compensate for functional defects by enhancing the synchrony of neural activity; on the other hand, with the decline of prefrontal executive control ability, the temporal lobe may compensate for the hypofunctional striatum-medial frontal cortex circuit, which is consistent with previous research by Li (Lee et al., 2019).

The right fusiform gyrus is a component of the visual cortex that mediates the perception and processing of visual information, and is widely involved in high-level cognitive processes such as working memory and attention (Han et al., 2013). The fusiform gyrus and the inferior temporal gyrus both belong to the ventral visual pathway and constitute an important part of the core network for facial recognition (Nishida et al., 2025). Structural or functional abnormalities in this region may further impair prefrontal lobe-related cognitive functions (Brunyé et al., 2017). Multiple studies have confirmed functional coupling between the prefrontal lobe and the fusiform gyrus (Druzgal and D'Esposito, 2003; Buchsbaum et al., 2005), which jointly participate in the regulatory mechanism of visual working memory. Bouhali found via diffusion tensor imaging that the fusiform gyrus is connected to advanced language regions such as the anterior temporal lobe and prefrontal lobe through the inferior longitudinal fasciculus and inferior fronto-occipital fasciculus (Bouhali et al., 2014). In addition, Prvulovic observed enhanced functional activation of the fusiform gyrus in Alzheimer’s disease patients performing visuospatial processing tasks (Prvulovic et al., 2002). Wang also found hyperactivity of the fusiform gyrus in PD-MCI patients using the amplitude of low-frequency fluctuation (ALFF) method, and hypothesized that the spatial adjacency of the fusiform gyrus to the parahippocampal gyrus may enable it to play an important role in compensating for memory deficits caused by parahippocampal gyrus abnormalities in PD-MCI patients (Wang et al., 2018). The increased ReHo value in the right fusiform gyrus observed in this study suggests that this phenomenon may reflect a cerebral compensatory mechanism, in which the brain achieves cognitive functions by enhancing the efficiency of visual information transmission.

Cerebellar lobule VIII belongs to the posterior lobe of the cerebellum (Keren-Happuch et al., 2014), which is not only involved in motor regulation but also modulates cognitive functions. Studies have shown that cerebellar lobule VIII is associated with working memory. In addition, multiple imaging studies have confirmed extensive neural connections between the cerebellar posterior lobe and the cerebral cortex as well as cortical neural networks (Bordignon et al., 2021; He et al., 2024; Saadon-Grosman et al., 2024). The cerebellar posterior lobe modulates the prefrontal lobe—a key node of the DMN and ECN—and thus jointly participates in the regulation of cognitive networks (Chen and Zhang, 2024). The cerebello-cerebral circuit, composed of the corticopontocerebellar afferent pathway and the cerebellothalamostriatal-cerebral efferent pathway, is the anatomical basis for the cerebellum to regulate cognitive functions (Kamali et al., 2010; Sokolov et al., 2014; Schmahmann et al., 2019). Furthermore, scholars have found a large number of cholinergic neurons in the cerebellar cortex and deep nuclei (Barr et al., 2024; Wakuda et al., 2025). Van used cholinergic [18F] fluoroethoxybenzovesamicol positron emission tomography (PET) scanning and found upregulated cholinergic activity in the cerebellum of PD-NC patients, indicating that the cerebellum exerts a compensatory effect in maintaining cognitive function in the early stage of PD (van der Zee et al., 2022). Some studies have also shown that the ReHo value is positively correlated with the severity of PD, and after taking anti-Parkinson’s drugs, the ReHo value of the cerebellum will significantly decrease (Zeng et al., 2017). This study also reminds us that compared with the HC group, the PD-NC group has shown a decrease in cerebellar ReHo values (although it has not yet manifested as cognitive impairment); However, when it progresses to the PD-MCI stage, the ReHo value increases instead. This might reflect a comprehensive impairment of cerebellar function. Therefore, the increase in the ReHo value of the right cerebellar hemisphere in PD-MCI patients requires considering both pathological injury and compensatory prefrontal lobe possibilities simultaneously.

Currently, the diagnosis of PD-MCI primarily relies on clinical interviews and assessment scales (e.g., MoCA), lacking objective biological markers. This study demonstrated through ROC analysis that alterations in ReHo within the Cerebellar lobule VIII and left inferior temporal gyrus exhibit high discriminative power for identifying cognitive impairment (AUC > 0.9), suggesting their potential as auxiliary diagnostic tools. The identified abnormal ReHo patterns provide potential neuroimaging biomarkers for early detection of cognitive impairment. This finding holds significant clinical value: (1) Improving diagnostic accuracy: Cognitive impairment symptoms in some PD patients may be masked by motor manifestations (e.g., reduced facial expressions, bradykinesia), leading to misdiagnosis. Objective measurement of ReHo in the Cerebellar lobule VIII and left inferior temporal gyrus aids in distinguishing cognitive impairment from PD-NC. (2) Guiding personalized treatment: Neuroregulatory techniques such as transcranial magnetic stimulation (TMS) and deep brain stimulation (DBS) have shown potential in treating cognitive impairment. Targeting these differential brain regions in our study may alleviate PD-MCI symptoms.

This study has several limitations. First, in the PD-MCI diagnostic guidelines issued by the MDS Task Force in 2012, Level II criteria require impairment in at least one neuropsychological test in each of two cognitive subdomains, and note that Level I criteria cannot fully classify the subtypes of PD-MCI. This study adopted Level I criteria, using the MoCA as a cognitive assessment tool. Although this scale covers multiple cognitive items, its total score is primarily used to screen for overall cognitive impairment, and its subitems suffer from insufficient reliability and validity when analyzed individually. Therefore, no systematic assessment or classification of cognitive subdomains was performed. Second, as the ROC analysis is exploratory and lacks an independent validation cohort, the reported diagnostic accuracy (AUC) is likely optimistic. To confirm these preliminary findings and rigorously evaluate their biomarker potential, future studies should employ larger, multi-center cohorts and longitudinal designs incorporating multimodal neuroimaging and robust analytical methods (e.g., voxel-wise correlation and training–testing validation). Furthermore, conducting detailed subdomain analyses of cognitive scales are essential to provide a comprehensive and objective basis for elucidating the pathogenesis of PD-MCI.

## 5 Conclusion

Based on the ReHo method of rs-fMRI, this study found that PD-MCI patients have abnormal local neural synchrony in multiple brain regions including the prefrontal lobe, temporal lobe, fusiform gyrus and cerebellum, and these abnormalities are significantly correlated with the degree of cognitive impairment. It was also found that posterior cortical brain regions may compensate for executive function deficits caused by damage to the striatal circuit. The above brain regions may serve as potential imaging markers for the early identification of PD-MCI. Resting-state ReHo technology can be used as an effective method to study the mechanism of cognitive impairment in PD patients, thereby helping to monitor the cognitive impairment status of PD patients and provide a basis for early intervention and treatment.

## Data Availability

The original contributions presented in the study are included in the article/supplementary material, further inquiries can be directed to the corresponding author/s.

## Footnotes

1
http://www.restfmri.net/forum/restplus
